# 2-[3-Hy­droxy-4-(2-hy­droxy­eth­oxy)phen­yl]-4,4,5,5-tetra­methyl-2-imidazoline-1-oxyl 3-oxide

**DOI:** 10.1107/S1600536811053979

**Published:** 2011-12-23

**Authors:** Hui-Ping Ma, Lin-Lin Jing, Lei He, Peng-Cheng Fan, Zheng-Ping Jia

**Affiliations:** a Department of Pharmacy, Lanzhou General Hospital of PLA, Key Laboratory of the Prevention and Cure for the Plateau Environment Damage, PLA 730050, Lanzhou Gansu, People’s Republic of China

## Abstract

In the title compound, C_15_H_21_N_2_O_5_, the imidazoline ring displays a twisted conformation. The mean plane of the imidazoline ring makes a dihedral angle of 22.55 (5)° with the benzene ring. In the crystal, O—H⋯O and C—H⋯O hydrogen bonds link the mol­ecules into a layer parallel to the *bc* plane.

## Related literature

For the biological activity of nitronyl nitroxides, see: Soule *et al.* (2007 ▶); Blasig *et al.* (2002 ▶); Qin *et al.* (2009 ▶); Tanaka *et al.* (2007 ▶). For puckering parameters, see: Cremer & Pople (1975 ▶). For pseudorotation parameters, see: Rao *et al.* (1981 ▶). For related structures, see: Jing, Ma, Fan *et al.* (2011 ▶); Jing, Ma, He *et al.* (2011 ▶). 
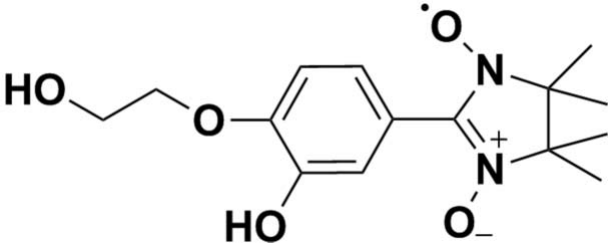


## Experimental

### 

#### Crystal data

C_15_H_21_N_2_O_5_
*M* *_r_* = 309.34
Monoclinic, 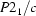

*a* = 9.787 (4) Å
*b* = 9.302 (3) Å
*c* = 16.657 (6) Å
β = 93.525 (3)°
*V* = 1513.5 (10) Å^3^
*Z* = 4
Mo *K*α radiation
μ = 0.10 mm^−1^
*T* = 296 K
0.25 × 0.23 × 0.21 mm

#### Data collection

Bruker APEXII CCD diffractometer
Absorption correction: multi-scan (*SADABS*; Bruker, 2007 ▶) *T*_min_ = 0.975, *T*_max_ = 0.979
7023 measured reflections
2784 independent reflections
2172 reflections with *I* > 2σ(*I*)
*R*_int_ = 0.023

#### Refinement

*R*[*F* ^2^ > 2σ(*F* ^2^)] = 0.038
*wR*(*F* ^2^) = 0.103
*S* = 1.01
2784 reflections
206 parameters
H-atom parameters constrained
Δρ_max_ = 0.21 e Å^−3^
Δρ_min_ = −0.14 e Å^−3^

### 

Data collection: *APEX2* (Bruker, 2007 ▶); cell refinement: *SAINT* (Bruker, 2007 ▶); data reduction: *SAINT*; program(s) used to solve structure: *SHELXS97* (Sheldrick, 2008 ▶); program(s) used to refine structure: *SHELXL97* (Sheldrick, 2008 ▶); molecular graphics: *ORTEP-3* (Farrugia, 1997 ▶); software used to prepare material for publication: *SHELXTL* (Sheldrick, 2008 ▶) and *PLATON* (Spek, 2009 ▶).

## Supplementary Material

Crystal structure: contains datablock(s) I, global. DOI: 10.1107/S1600536811053979/is5030sup1.cif

Structure factors: contains datablock(s) I. DOI: 10.1107/S1600536811053979/is5030Isup2.hkl

Additional supplementary materials:  crystallographic information; 3D view; checkCIF report

## Figures and Tables

**Table 1:** Hydrogen-bond geometry (Å, °)

*D*—H⋯*A*	*D*—H	H⋯*A*	*D*⋯*A*	*D*—H⋯*A*
O3—H3*A*⋯O1^i^	0.82	1.91	2.678 (2)	156
O5—H5*A*⋯O3^ii^	0.82	2.34	2.993 (2)	137
O5—H5*A*⋯O4^ii^	0.82	2.46	3.111 (2)	137
C12—H12*A*⋯O5^iii^	0.96	2.57	3.410 (3)	146

Symmetry codes: (i) 
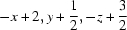
; (ii) 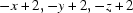; (iii) 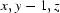.

**Fig. 1.**
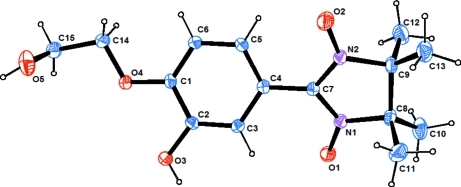
Molecular structure of the title compound with atom numbering scheme. Displacement ellipsoids are drawn at the 30% probability level. H atoms are presented as a small spheres of arbitrary radius.

## supplementary crystallographic information

### Comment

Nitronyl nitroxides, which can react with free radicals such as OH, H_2_O_2_, and O_2_ (Blasig *et al.*, 2002) to protect cells from the attack of free radicals have lots of biological properities as anticancer, antiradiation and antioxidation (Qin *et al.*, 2009; Tanaka *et al.*, 2007; Soule *et al.*, 2007).

The molecular structure of the title compound is shown in Fig. 1. The nitronyl nitroxide ring and the phenyl rings are twisted with respect to each other making a dihedral angle of 22.55 (5)°. The puckering parameters of the nitronyl nitroxide ring are Q(2) = 0.2645 (17) Å and φ = 121.9 (4)° (Cremer & Pople, 1975). The pseudorotation parameters (Rao *et al.*, 1981) for the nitronyl nitroxide ring are P = 283.2 (2)° and τ(*M*) = 27.1 (1)° for the C7—N1 reference bond with the closest puckering descriptor being twisted on C8—C9. The crystal structure is stabilized by O—H···O and C—H···O hydrogen bonds (Table 1).

### Experimental

To a solution of 3-hydroxy-4-(2-hydroxyethoxy)benzaldehyde (0.91 g, 5 mmol) in methanol (20 ml), 2,3-dimethyl-2,3-bis(hydroxylamino) butane (0.74 g, 5.0 mmol) was added. The mixture was stirring for 24 h at room temperature then filtered. The resulting white powder was suspended in the solution of dichloromethane (20.0 ml). An aqueous solution of NaIO_4_ (20 ml) was added to the reaction mixture and stirred for 15 min in an ice bath. The aqueous phase was extracted with CH_2_Cl_2_ and the combined organic layers were washed with brine (20 ml) and dried over Na_2_SO_4_. the solvent was removed to obtain a dark blue residue which was purified by flash column chromatography with the elution of dichloromethane/ methanol (10:1) to yield 0.70 g (45%) of the title compound as a dark blue powder. Single crystals of the title compound suitable for X-ray diffraction was recrystallized from hexane/dichloromethane (1:2).

### Refinement

H atoms were positioned geometrically and refined using a riding model, with C—H_methyl_ = 0.96 Å, C—H_methylene_ = 0.97 Å, C—H_aryl_ = 0.93 Å and O—H = 0.82 Å, and with *U*_iso_(H) = 1.2*U*_eq_(C) or 1.5*U*_eq_(C_methyl_).

### Crystal data

**Table:**

C_15_H_21_N_2_O_5_	*F*(000) = 660
*M**_r_* = 309.34	*D*_x_ = 1.358 Mg m^−^^3^
Monoclinic, *P*2_1_/*c*	Mo *K*α radiation, λ = 0.71073 Å
Hall symbol: -P 2ybc	Cell parameters from 2979 reflections
*a* = 9.787 (4) Å	θ = 2.5–27.7°
*b* = 9.302 (3) Å	µ = 0.10 mm^−^^1^
*c* = 16.657 (6) Å	*T* = 296 K
β = 93.525 (3)°	Block, blue
*V* = 1513.5 (10) Å^3^	0.25 × 0.23 × 0.21 mm
*Z* = 4	

### Data collection

**Table:**

Bruker APEXII CCD diffractometer	2784 independent reflections
Radiation source: fine-focus sealed tube	2172 reflections with *I* > 2σ(*I*)
graphite	*R*_int_ = 0.023
φ and ω scans	θ_max_ = 25.5°, θ_min_ = 2.5°
Absorption correction: multi-scan (*SADABS*; Bruker, 2007)	*h* = −11→8
*T*_min_ = 0.975, *T*_max_ = 0.979	*k* = −11→10
7023 measured reflections	*l* = −20→19

### Refinement

**Table:**

Refinement on *F*^2^	Secondary atom site location: difference Fourier map
Least-squares matrix: full	Hydrogen site location: inferred from neighbouring sites
*R*[*F*^2^ > 2σ(*F*^2^)] = 0.038	H-atom parameters constrained
*wR*(*F*^2^) = 0.103	*w* = 1/[σ^2^(*F*_o_^2^) + (0.0486*P*)^2^ + 0.519*P*] where *P* = (*F*_o_^2^ + 2*F*_c_^2^)/3
*S* = 1.01	(Δ/σ)_max_ < 0.001
2784 reflections	Δρ_max_ = 0.21 e Å^−^^3^
206 parameters	Δρ_min_ = −0.14 e Å^−^^3^
0 restraints	Extinction correction: *SHELXL97* (Sheldrick, 2008), Fc^*^=kFc[1+0.001xFc^2^λ^3^/sin(2θ)]^-1/4^
Primary atom site location: structure-invariant direct methods	Extinction coefficient: 0.0167 (18)

### Special details

**Table:**

Geometry. All e.s.d.'s (except the e.s.d. in the dihedral angle between two l.s. planes) are estimated using the full covariance matrix. The cell e.s.d.'s are taken into account individually in the estimation of e.s.d.'s in distances, angles and torsion angles; correlations between e.s.d.'s in cell parameters are only used when they are defined by crystal symmetry. An approximate (isotropic) treatment of cell e.s.d.'s is used for estimating e.s.d.'s involving l.s. planes.
Refinement. Refinement of *F*^2^ against ALL reflections. The weighted *R*-factor *wR* and goodness of fit *S* are based on *F*^2^, conventional *R*-factors *R* are based on *F*, with *F* set to zero for negative *F*^2^. The threshold expression of *F*^2^ > σ(*F*^2^) is used only for calculating *R*-factors(gt) *etc*. and is not relevant to the choice of reflections for refinement. *R*-factors based on *F*^2^ are statistically about twice as large as those based on *F*, and *R*- factors based on ALL data will be even larger.

### Fractional atomic coordinates and isotropic or equivalent isotropic displacement parameters (Å^2^)

**Table:**

	*x*	*y*	*z*	*U*_iso_*/*U*_eq_	
C1	0.84569 (17)	0.65658 (17)	0.96445 (9)	0.0307 (4)	
C2	0.90504 (16)	0.61269 (17)	0.89405 (9)	0.0291 (4)	
C3	0.86536 (16)	0.48613 (17)	0.85697 (9)	0.0294 (4)	
H3	0.9052	0.4577	0.8103	0.035*	
C4	0.76514 (16)	0.39970 (17)	0.88925 (9)	0.0287 (4)	
C5	0.70732 (19)	0.44374 (19)	0.95909 (10)	0.0399 (4)	
H5	0.6409	0.3870	0.9812	0.048*	
C6	0.74747 (19)	0.57120 (19)	0.99621 (10)	0.0403 (5)	
H6	0.7078	0.5995	1.0430	0.048*	
C7	0.71796 (16)	0.26786 (17)	0.84938 (9)	0.0276 (4)	
C8	0.69965 (17)	0.06957 (17)	0.76020 (10)	0.0330 (4)	
C9	0.58253 (17)	0.06193 (17)	0.81818 (10)	0.0330 (4)	
C10	0.7809 (2)	−0.0678 (2)	0.75295 (14)	0.0548 (6)	
H10A	0.8528	−0.0526	0.7172	0.082*	
H10B	0.7214	−0.1428	0.7320	0.082*	
H10C	0.8199	−0.0952	0.8050	0.082*	
C11	0.6547 (2)	0.1276 (2)	0.67686 (11)	0.0498 (5)	
H11A	0.6049	0.2156	0.6824	0.075*	
H11B	0.5972	0.0582	0.6487	0.075*	
H11C	0.7340	0.1456	0.6471	0.075*	
C12	0.6080 (2)	−0.0420 (2)	0.88796 (12)	0.0538 (5)	
H12A	0.6964	−0.0235	0.9141	0.081*	
H12B	0.6050	−0.1389	0.8681	0.081*	
H12C	0.5387	−0.0294	0.9257	0.081*	
C13	0.44130 (18)	0.0385 (2)	0.77753 (12)	0.0476 (5)	
H13A	0.3753	0.0308	0.8176	0.071*	
H13B	0.4411	−0.0484	0.7464	0.071*	
H13C	0.4181	0.1183	0.7428	0.071*	
C14	0.8384 (2)	0.83103 (19)	1.06967 (10)	0.0397 (4)	
H14A	0.8655	0.7640	1.1123	0.048*	
H14B	0.7392	0.8347	1.0644	0.048*	
C15	0.8949 (2)	0.9769 (2)	1.08947 (11)	0.0419 (5)	
H15A	0.8817	0.9993	1.1453	0.050*	
H15B	0.9924	0.9784	1.0819	0.050*	
N1	0.78767 (13)	0.18428 (14)	0.80059 (8)	0.0290 (3)	
N2	0.59145 (14)	0.20965 (14)	0.85370 (8)	0.0328 (3)	
O1	0.91259 (12)	0.20380 (13)	0.78261 (7)	0.0408 (3)	
O2	0.49072 (13)	0.26599 (15)	0.88724 (9)	0.0549 (4)	
O3	1.00204 (13)	0.70239 (14)	0.86708 (8)	0.0450 (4)	
H3A	1.0205	0.6781	0.8217	0.067*	
O4	0.89083 (12)	0.78448 (12)	0.99533 (7)	0.0377 (3)	
O5	0.82812 (16)	1.08039 (14)	1.03935 (10)	0.0603 (4)	
H5A	0.8753	1.1532	1.0385	0.090*	

### Atomic displacement parameters (Å^2^)

**Table:**

	*U*^11^	*U*^22^	*U*^33^	*U*^12^	*U*^13^	*U*^23^
C1	0.0359 (9)	0.0251 (8)	0.0311 (8)	−0.0062 (7)	0.0024 (7)	−0.0044 (7)
C2	0.0272 (8)	0.0273 (8)	0.0335 (8)	−0.0041 (7)	0.0065 (7)	0.0005 (7)
C3	0.0293 (9)	0.0290 (9)	0.0306 (8)	−0.0009 (7)	0.0069 (7)	−0.0044 (7)
C4	0.0304 (9)	0.0261 (8)	0.0297 (8)	−0.0029 (7)	0.0026 (7)	−0.0020 (6)
C5	0.0500 (11)	0.0345 (10)	0.0366 (9)	−0.0179 (8)	0.0149 (8)	−0.0064 (8)
C6	0.0525 (12)	0.0372 (10)	0.0330 (9)	−0.0155 (9)	0.0182 (8)	−0.0096 (7)
C7	0.0282 (9)	0.0256 (8)	0.0294 (8)	−0.0014 (7)	0.0039 (7)	−0.0020 (6)
C8	0.0319 (9)	0.0275 (9)	0.0393 (9)	−0.0010 (7)	0.0008 (7)	−0.0103 (7)
C9	0.0349 (9)	0.0257 (9)	0.0383 (9)	−0.0058 (7)	0.0001 (7)	−0.0077 (7)
C10	0.0427 (12)	0.0354 (11)	0.0864 (16)	0.0029 (9)	0.0069 (11)	−0.0212 (10)
C11	0.0516 (12)	0.0604 (13)	0.0370 (10)	−0.0061 (10)	0.0010 (9)	−0.0106 (9)
C12	0.0684 (15)	0.0405 (11)	0.0520 (12)	−0.0144 (10)	−0.0018 (10)	0.0039 (9)
C13	0.0332 (10)	0.0472 (11)	0.0619 (12)	−0.0073 (9)	−0.0003 (9)	−0.0173 (10)
C14	0.0469 (11)	0.0383 (10)	0.0350 (9)	−0.0117 (8)	0.0114 (8)	−0.0096 (7)
C15	0.0455 (11)	0.0407 (11)	0.0397 (9)	−0.0105 (9)	0.0048 (8)	−0.0124 (8)
N1	0.0261 (7)	0.0283 (7)	0.0329 (7)	−0.0013 (6)	0.0037 (6)	−0.0048 (6)
N2	0.0287 (8)	0.0308 (8)	0.0397 (8)	−0.0041 (6)	0.0086 (6)	−0.0106 (6)
O1	0.0285 (7)	0.0448 (8)	0.0504 (7)	−0.0046 (5)	0.0128 (5)	−0.0120 (6)
O2	0.0346 (7)	0.0536 (9)	0.0789 (10)	−0.0084 (6)	0.0227 (7)	−0.0303 (7)
O3	0.0498 (8)	0.0398 (7)	0.0478 (8)	−0.0199 (6)	0.0229 (6)	−0.0127 (6)
O4	0.0459 (7)	0.0306 (6)	0.0380 (7)	−0.0130 (5)	0.0142 (5)	−0.0105 (5)
O5	0.0572 (9)	0.0358 (8)	0.0862 (11)	−0.0076 (7)	−0.0097 (8)	−0.0098 (7)

### Geometric parameters (Å, °)

**Table:**

C1—O4	1.3591 (19)	C10—H10B	0.9600
C1—C6	1.377 (2)	C10—H10C	0.9600
C1—C2	1.401 (2)	C11—H11A	0.9600
C2—O3	1.3607 (19)	C11—H11B	0.9600
C2—C3	1.374 (2)	C11—H11C	0.9600
C3—C4	1.401 (2)	C12—H12A	0.9600
C3—H3	0.9300	C12—H12B	0.9600
C4—C5	1.387 (2)	C12—H12C	0.9600
C4—C7	1.456 (2)	C13—H13A	0.9600
C5—C6	1.383 (2)	C13—H13B	0.9600
C5—H5	0.9300	C13—H13C	0.9600
C6—H6	0.9300	C14—O4	1.436 (2)
C7—N1	1.341 (2)	C14—C15	1.494 (2)
C7—N2	1.357 (2)	C14—H14A	0.9700
C8—N1	1.504 (2)	C14—H14B	0.9700
C8—C10	1.514 (2)	C15—O5	1.408 (2)
C8—C11	1.529 (3)	C15—H15A	0.9700
C8—C9	1.545 (2)	C15—H15B	0.9700
C9—N2	1.496 (2)	N1—O1	1.2893 (17)
C9—C13	1.517 (2)	N2—O2	1.2753 (18)
C9—C12	1.521 (3)	O3—H3A	0.8200
C10—H10A	0.9600	O5—H5A	0.8200
			
O4—C1—C6	125.29 (15)	C8—C11—H11A	109.5
O4—C1—C2	115.46 (14)	C8—C11—H11B	109.5
C6—C1—C2	119.24 (14)	H11A—C11—H11B	109.5
O3—C2—C3	124.08 (14)	C8—C11—H11C	109.5
O3—C2—C1	115.55 (14)	H11A—C11—H11C	109.5
C3—C2—C1	120.37 (14)	H11B—C11—H11C	109.5
C2—C3—C4	120.29 (14)	C9—C12—H12A	109.5
C2—C3—H3	119.9	C9—C12—H12B	109.5
C4—C3—H3	119.9	H12A—C12—H12B	109.5
C5—C4—C3	118.94 (15)	C9—C12—H12C	109.5
C5—C4—C7	119.81 (14)	H12A—C12—H12C	109.5
C3—C4—C7	121.21 (14)	H12B—C12—H12C	109.5
C6—C5—C4	120.59 (15)	C9—C13—H13A	109.5
C6—C5—H5	119.7	C9—C13—H13B	109.5
C4—C5—H5	119.7	H13A—C13—H13B	109.5
C1—C6—C5	120.57 (15)	C9—C13—H13C	109.5
C1—C6—H6	119.7	H13A—C13—H13C	109.5
C5—C6—H6	119.7	H13B—C13—H13C	109.5
N1—C7—N2	107.49 (13)	O4—C14—C15	108.50 (14)
N1—C7—C4	127.28 (14)	O4—C14—H14A	110.0
N2—C7—C4	125.20 (14)	C15—C14—H14A	110.0
N1—C8—C10	110.22 (14)	O4—C14—H14B	110.0
N1—C8—C11	106.28 (14)	C15—C14—H14B	110.0
C10—C8—C11	110.42 (16)	H14A—C14—H14B	108.4
N1—C8—C9	100.34 (12)	O5—C15—C14	109.76 (15)
C10—C8—C9	115.11 (15)	O5—C15—H15A	109.7
C11—C8—C9	113.64 (15)	C14—C15—H15A	109.7
N2—C9—C13	109.74 (14)	O5—C15—H15B	109.7
N2—C9—C12	106.19 (14)	C14—C15—H15B	109.7
C13—C9—C12	110.61 (16)	H15A—C15—H15B	108.2
N2—C9—C8	100.22 (12)	O1—N1—C7	125.74 (13)
C13—C9—C8	114.72 (14)	O1—N1—C8	121.41 (12)
C12—C9—C8	114.44 (15)	C7—N1—C8	112.56 (13)
C8—C10—H10A	109.5	O2—N2—C7	126.26 (14)
C8—C10—H10B	109.5	O2—N2—C9	121.51 (13)
H10A—C10—H10B	109.5	C7—N2—C9	112.12 (13)
C8—C10—H10C	109.5	C2—O3—H3A	109.5
H10A—C10—H10C	109.5	C1—O4—C14	117.73 (12)
H10B—C10—H10C	109.5	C15—O5—H5A	109.5
			
O4—C1—C2—O3	1.4 (2)	C11—C8—C9—C12	158.75 (15)
C6—C1—C2—O3	−179.41 (16)	O4—C14—C15—O5	74.74 (19)
O4—C1—C2—C3	−178.96 (15)	N2—C7—N1—O1	−178.94 (14)
C6—C1—C2—C3	0.2 (3)	C4—C7—N1—O1	2.8 (3)
O3—C2—C3—C4	179.57 (16)	N2—C7—N1—C8	7.18 (18)
C1—C2—C3—C4	0.0 (2)	C4—C7—N1—C8	−171.10 (15)
C2—C3—C4—C5	−0.2 (2)	C10—C8—N1—O1	42.6 (2)
C2—C3—C4—C7	177.74 (15)	C11—C8—N1—O1	−77.09 (18)
C3—C4—C5—C6	0.2 (3)	C9—C8—N1—O1	164.37 (14)
C7—C4—C5—C6	−177.73 (16)	C10—C8—N1—C7	−143.25 (16)
O4—C1—C6—C5	178.91 (17)	C11—C8—N1—C7	97.09 (16)
C2—C1—C6—C5	−0.2 (3)	C9—C8—N1—C7	−21.46 (17)
C4—C5—C6—C1	−0.1 (3)	N1—C7—N2—O2	−172.08 (16)
C5—C4—C7—N1	−155.39 (17)	C4—C7—N2—O2	6.2 (3)
C3—C4—C7—N1	26.7 (2)	N1—C7—N2—C9	11.58 (18)
C5—C4—C7—N2	26.6 (3)	C4—C7—N2—C9	−170.10 (15)
C3—C4—C7—N2	−151.31 (16)	C13—C9—N2—O2	38.3 (2)
N1—C8—C9—N2	24.92 (14)	C12—C9—N2—O2	−81.3 (2)
C10—C8—C9—N2	143.18 (15)	C8—C9—N2—O2	159.39 (15)
C11—C8—C9—N2	−88.10 (16)	C13—C9—N2—C7	−145.15 (15)
N1—C8—C9—C13	142.35 (15)	C12—C9—N2—C7	95.28 (17)
C10—C8—C9—C13	−99.39 (19)	C8—C9—N2—C7	−24.07 (16)
C11—C8—C9—C13	29.3 (2)	C6—C1—O4—C14	4.0 (3)
N1—C8—C9—C12	−88.24 (16)	C2—C1—O4—C14	−176.92 (15)
C10—C8—C9—C12	30.0 (2)	C15—C14—O4—C1	−176.62 (15)

### Hydrogen-bond geometry (Å, °)

**Table:**

*D*—H···*A*	*D*—H	H···*A*	*D*···*A*	*D*—H···*A*
O3—H3A···O1^i^	0.82	1.91	2.678 (2)	156
O5—H5A···O3^ii^	0.82	2.34	2.993 (2)	137
O5—H5A···O4^ii^	0.82	2.46	3.111 (2)	137
C12—H12A···O5^iii^	0.96	2.57	3.410 (3)	146

Symmetry codes: (i) −*x*+2, *y*+1/2, −*z*+3/2; (ii) −*x*+2, −*y*+2, −*z*+2; (iii) *x*, *y*−1, *z*.
